# Definition of Scenarios for Modern Power Systems with a High Renewable Energy Share

**DOI:** 10.1002/gch2.202200129

**Published:** 2023-02-28

**Authors:** Carlos Collados‐Rodríguez, Eduard Antolí‐Gil, Enric Sánchez‐Sánchez, Jaume Girona‐Badia, Vinicius Albernaz Lacerda, Marc Cheah‐Mañe, Eduardo Prieto‐Araujo, Oriol Gomis‐Bellmunt

**Affiliations:** ^1^ CITCEA‐UPC Av Diagonal 647, H building, 2nd floor Barcelona Spain

**Keywords:** future power systems, future scenarios, generation mix, optimization, renewable energy sources

## Abstract

Recent environmental policies have led academic, industrial, and governmental stakeholders to plan scenarios with a high share of renewable energy sources (RES), to ensure that future energy systems, composed mostly of RES, can remain stable, match the demand during seasonal variations and are economically feasible. This article considers different energy scenarios to obtain various options in terms of size, generation technologies, and grid configuration. The scenarios are studied in the POSYTYF project and are quantified through an optimization‐based algorithm, where the test grids topologies are based on specific locations in Europe, and real data related to the availability of RES, as well as the demand. Different RES technologies are considered to meet requirements of grid integration of renewables at different horizons of time, up to 100% in the most futuristic case. The optimization algorithm is applied to three scenarios. It is shown that solar photovoltaic (PV) and wind can provide the renewable backbone, but they lack flexibility to achieve a very high share in the energy mix. Solar thermal and pumped hydro become important to cover the last range of integration, as they provide high flexibility, which is crucial for high share, but they are expensive for low share.

## Graphical abstract

Recent environmental policies have led to increasing planning of scenarios with a high share of renewable energy sources (RES), to ensure that future RES‐based systems can remain stable, match the demand during seasonal variations, and are economically feasible. This article considers different energy scenarios to obtain various options in terms of size, generation technologies, and grid configuration.

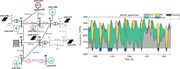

## 1 Introduction

The electrical power system is experiencing a deep transformation worldwide, due to the massive integration of renewable energy, the electrification of the demand, and the irruption of electric mobility. This trend is intensifying, and power systems have to achieve a massive share of renewable energy in the next decades. These dramatic changes impose new challenges to the systems’ stability and control, due to the reduction of inertia and short‐circuit levels.^[^ ^2^ ^]^ Recently, several countries have defined targets to reduce the participation of fossil‐fuel‐based sources in the energy mix while increasing the integration of Renewable Energy Sources (RES), such as wind, solar, geothermal, hydro, ocean and biomass. In the same direction, the European Union has set targets for specific levels of RES integration in the future European energy mix, with progressive participation of 20% in 2020,^[^ ^28^ ^]^ 32% in 2030 and two‐thirds in 2050.^[^ ^24^, ^25^, ^27^ ^]^ These goals are to be achieved considering the participation of all Member States, which are defining their own policies and goals to match the general targets. For instance, Spain has established a target of 42% of RES share on energy end‐use by 2030.^[^ ^74^ ^]^ Germany and France defined a target of 65% and 40% of RES in the final electricity consumption, respectively.^[^ ^26^ ^]^

In order to achieve the aforementioned targets, realistic power systems models are required, considering a variety of technologies, system topologies, and elements such as high‐voltage direct current (HVDC),^[^ ^78^ ^]^ microgrids,^[^ ^34^ ^]^ virtual power plants, and dynamic virtual power plants.^[^ ^53^ ^]^ Various future scenarios are being analyzed for each system and region to ensure that the future energy systems, composed mostly of RES, can remain stable, reliable, match the demand during the seasonal variations across the year, and are economically feasible. These studies are regional by nature as they consider local weather and the availability of resources. Some examples were conducted for provinces or regions, such as Ontario,^[^ ^55^ ^]^ British Columbia,^[^ ^63^ ^]^ the New York State,^[^ ^51^ ^]^ among others.^[^ ^7^, ^13^, ^31^, ^46^ ^]^ Similar studies have also been performed using data from countries, such as Australia,^[^ ^15^ ^]^ Bangladesh,^[^ ^33^ ^]^ Brazil,^[^ ^14^, ^72^ ^]^ Chile,^[^ ^54^ ^]^ France,^[^ ^47^ ^]^ Germany,^[^ ^66^ ^]^ India,^[^ ^4^ ^]^ Italy, Pakistan,^[^ ^71^ ^]^ Portugal,^[^ ^30^, ^65^ ^]^ United Arab Emirates,^[^ ^3^ ^]^ and the United States.^[^ ^52^ ^]^ Other studies have also analyzed systems with an ambitious goal of 100% of RES.^[^ ^36^, ^50^, ^58^, ^62^, ^81^ ^]^ Moreover, as the number of studies has largely increased, several tools have been proposed to assist the generation expansion planning and RES design, such as EnergyPLAN,^[^ ^22^ ^]^ EnergyScopeTD,^[^ ^49^ ^]^ HOMER,^[^ ^37^ ^]^ LEAP,^[^ ^75^ ^]^ SILVER,^[^ ^55^ ^]^ TIMES,^[^ ^39^ ^]^ among others.^[^ ^11^ ^]^ However, most of these studies focus on renewable resources while keeping the electrical grid out of their analysis. The contrary is also true for the grids, as several power system benchmarks have been proposed without a clear rationale for the resource type and location in the grid. A few examples are the IEEE and the CIGRE benchmarks.^[^ ^64^ ^]^ As the power systems are adapting to support a massive integration of RES, both the resources and the grid play a key role when defining modern power system scenarios. These scenarios should be prepared with optimization techniques, where the proper choice is unclear as there are numerous different methods and approaches.^[^ ^56^ ^]^

In this direction, the present overview summarizes several generation technologies and defines relevant future scenarios capturing the key features of the different renewable energy generation technologies, geographic and demand considerations, and electrical topologies. The future scenarios were defined in the context of the POSYTYF project.^[^ ^67^ ^]^ The presented concepts can be used as a starting point to conduct more detailed other studies on different representative scenarios. Aspects related to cost, efficiency, resource availability, and flexibility of different generation technologies are considered. Moreover, an optimization methodology is used to size the renewable power plants in different example scenarios, considering cost, and availability. Therefore, this overview helps to understand the benefits of combining a wide range of different renewable energy generation technologies, where some provide generation at low cost but are not controllable, while others provide more controllability at a higher cost, but are fundamental for massive integration of renewables. The conclusions of our study are in agreement with the most recent studies on the future 100% RES‐based systems, as comprehensively shown in Breyer et al.^[^ ^8^ ^]^

Overall the paper provides insights to define future power systems scenarios with high penetration of RES that might help to conduct further studies. Moreover, further contributions can be summarized as follows:

- It presents a comprehensive revision of existing renewable generation technologies, classifying them according to the controllability and flexibility they can provide in modern power grids.
- It defines conceptually some possible electrical layouts that can be found in modern power systems, including the need for HVDC transmission systems.
- It suggests a methodology to size the power plants for given scenarios, in order to complete the scenario definition and be able to specify some specific scenarios of modern power systems.

The remainder of this overview is organized as follows. Section 2 briefly introduces each generation technology. Section 3 presents generated scenarios. Section 4 describes the methodology used to size the scenarios, including the optimization algorithm. Section 5 presents the defined scenarios resulting from the optimization algorithm. Finally, the conclusions are drawn in Section 6.

## 2 Generation Technologies

This section presents an overview of the most relevant renewable and conventional generation technologies, highlighting different characteristics that must be considered for an adequate sizing of the generation mix, with examples presented in the following sections.

### 2.1 Solar Photovoltaic

**Figure 1:**
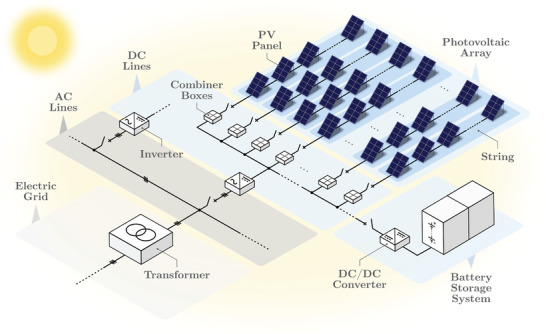
General scheme of a photovoltaic (PV) power plant.

Photovoltaic (PV) systems encompass several PV modules (**Figure** **1**). These modules are characterized by the well‐known *I*–*V* curve, which depends on external conditions such as solar radiation levels and temperature. In order to obtain the maximum power output, the module must work as near as possible to the maximum power point (MPP), which is close to the knee of the *I*–*V* characteristic curve. For this purpose, power electronic devices such as inverters constantly track the MPP considering solar radiation and temperature variations. Furthermore, these are employed for DC/AC conversion to connect the PV system to the grid. Although PV modules have negligible inherent storage capability, this can be provided by external devices.

### 2.2 Solar Thermal

**Figure 2:**
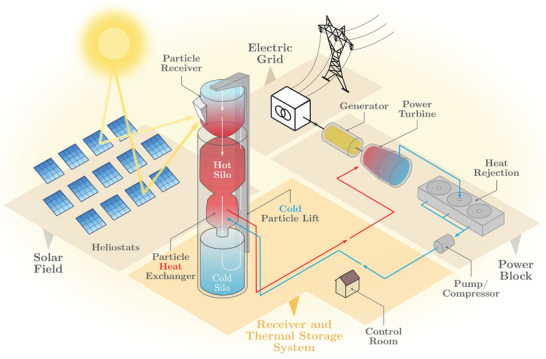
General scheme of a solar thermal power plant (solar tower).

Solar thermal technologies use solar concentrators to produce the required high temperatures in the working fluid to raise steam to drive heat engines, mainly turbines in commercial plants. Therefore, solar concentrators perform a function similar to a conventional thermal power plant boiler based on a Rankine cycle. Steam temperature is critical to obtain acceptable conversion efficiencies. Nowadays, three proven technologies, which require direct or beam radiation, are appropriate for a large‐scale generation: parabolic troughs or linear Fresnel reflectors (both corresponding to linear focus technology), solar towers (**Figure** **2**), or dishes (point focus technology). Depending on design details, large capacity thermal energy storage can be implemented, for instance, through molten salts. The inherent storage time is between 0 and 24 h.^[^ ^60^ ^]^

### 2.3 Wind

Wind air flow is established due to the pressure gradient between high‐pressure and low‐pressure zones, determining the initial speed and direction of wind flow.

**Figure 3:**
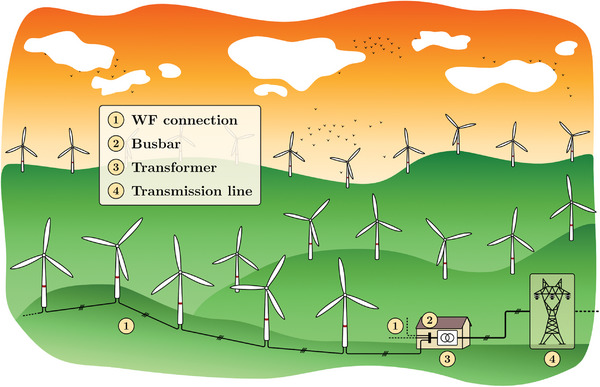
General scheme of an onshore wind farm.

**Figure 4:**
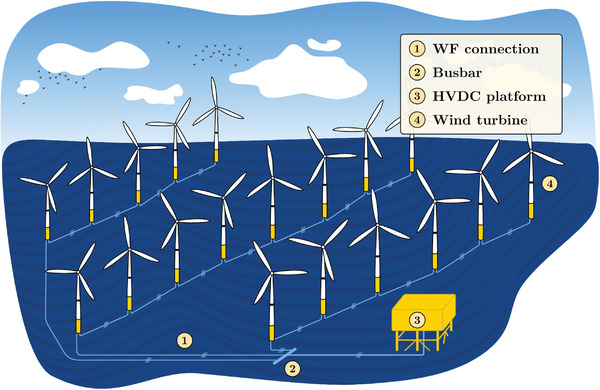
General scheme of an offshore wind farm.

Two types of wind farms can be distinguished: onshore wind farms (**Figure** **3**) and offshore wind farms (**Figure** **4**). Both types have several subsystems in common, such as AC connections between turbines, busbar, and transformer. Offshore wind farms might require exporting the generated power through HVDC technologies when these are located considerably far from shore (more than 80–100 km, approximately). Lastly, different grid topologies can be found depending on their interconnection, e.g., radial, ring or star configurations.^[^ ^78^ ^]^

### 2.4 Hydroelectric

**Figure 5:**
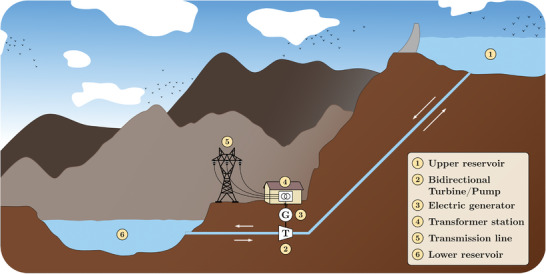
General scheme of a pumped‐storage hydropower plant (PS‐HPP).

Hydropower technologies take advantage of either water's potential or kinetic energy. Three main hydropower technologies can be distinguished: Impoundment or reservoir hydro facilities (created by damming rivers), diversion facilities or run‐of‐river hydropower (created by channeling a proportion of the river into a canal or penstock), and pumped‐storage hydropower plants (which have two water reservoirs) (PS‐HPPs) (**Figure** **5**).^[^ ^38^ ^]^ The suitability of each technology is highly dependent on the local topography.^[^ ^38^, ^40^ ^]^

In areas where the installation of large hydropower is unsuitable, PS‐HPP is a promising alternative to consider. A PS‐HPP comprises an upper and a lower reservoir and a binary or ternary pumping‐turbine set, as shown in Figure 5. Whenever electricity is needed, water is driven from the upper reservoir to the lower reservoir and electricity is generated via the turbine system. When there is a surplus of electricity generation, water stored in the lower reservoir can be pumped back to the upper reservoir.

### 2.5 Biomass

**Figure 6:**
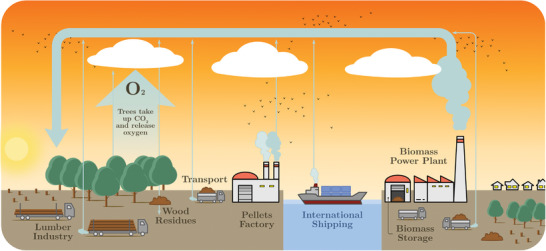
Conceptual scheme of the biomass resource process, including local generation and generation involving transport.

Biomass energy encompasses all sorts of solid biomass (such as wood and crops) or liquid biofuels that can be stored and used whenever required for electricity generation, similarly to fossil fuels, although with low energy density. If possible, biomass should be produced and consumed locally (see right‐hand side of **Figure** **6**). If the production and consumption are not local, the fuel required for transport will highly increase due to the low energy density of biomass. That is the reason why most biomass power plants rely on local feedstock and supply chains. Besides, their size is usually smaller than conventional power plants. Three thermochemical conversion technologies are distinguished in solid biomass: direct combustion, gasification, and pyrolysis.

### 2.6 Geothermal

**Figure 7:**
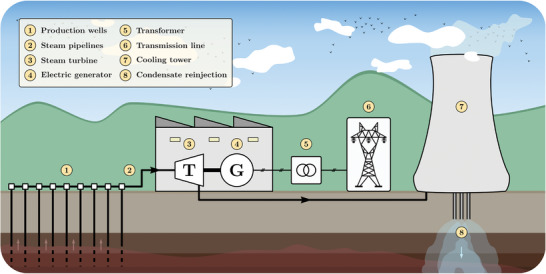
General scheme of a geothermal power plant.

Geothermal energy derives from heat within the subsurface of the earth.^[^ ^43^ ^]^ The heat transfer medium is water and/or steam. This renewable energy source is highly dependent on geographical locations. Besides electricity generation, heat can be used for heating greenhouses, buildings, or districts. Like other power plants, geothermal power plants use steam to drive steam turbines to produce electricity. A basic scheme of a generic geothermal power is shown in **Figure** **7**.

### 2.7 Thermal Coal/Fuel

**Figure 8:**
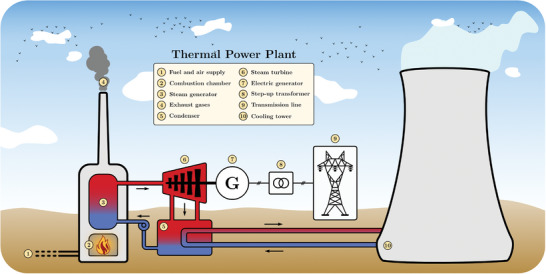
Conventional thermal power station.

Conventional thermal power plants which use fossil fuels to generate electricity are based on a Rankine cycle (**Figure** **8**). The coal/fuel burns inside the boiler, generating large amounts of heat used to produce highly pressurized steam. One or several sets of turbines (e.g., high, medium, or low pressure) generate rotating power via the steam mentioned above. Afterwards, the steam leaving the turbine's chamber is condensed using a cooling tower and recirculated back into the boiler to restart the cycle.^[^ ^16^ ^]^

### 2.8 Thermal Combined‐Cycle

**Figure 9:**
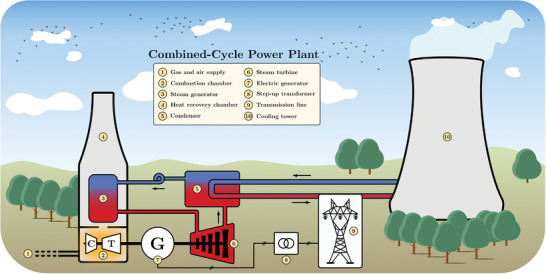
Combined‐cycle thermal power station.

Combined‐cycle power plants utilize natural gas to generate electricity (**Figure** **9**). The plant bases its operation on two thermodynamic cycles: the Brayton cycle (gas turbine) and the Rankine cycle (steam turbine). Regarding the gas cycle, external air is compressed to high pressure through a compressor and mixed with gas. Then, the combustion takes place, and the combustion gasses expand in the turbine. Finally, the exhaust gasses are driven to a recovery boiler to raise steam for the steam cycle. Usually, both turbines are coupled to the same shaft.^[^ ^17^ ^]^

These power plants have higher efficiencies than conventional thermal power plants and can operate at a broader range of powers (min. 45% of the rated power). Moreover, their greenhouse gas emissions and refrigerating water consumption are lower. Also, for the same installed capacity, the infrastructure footprint is smaller.

### 2.9 Nuclear

**Figure 10:**
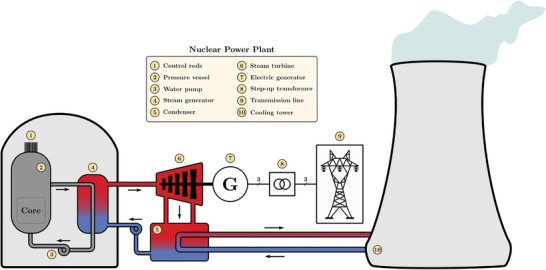
Pressurized water reactor (PWR) nuclear power plant.

The most common reactor in a nuclear power plant is the pressurized water reactor (PWR). **Figure** **10** sketches the main subsystems in a PWR nuclear power plant. Like thermal power plants utilizing fossil fuels, PWR plants are based on the Rankine cycle. However, in these power plants, fission produces heat in a reactor vessel containing water at very high pressure. Then, via a heat exchanger, the primary circuit transfers its energy to the secondary circuit.

### 2.10 Metrics Definition and Summary

The following concepts are defined to compare the different features of each technology:

- **Controllability**: Capability of a generation technology to store and control the power exchange with the network. The plant will be fully controllable if it can adjust the energy output and store the energy that is not currently used. Also, the larger the storage, the higher the controllability. Nevertheless, a plant with zero controllability cannot control the output power or can control the output power but cannot store the currently unused power. Level definitions:
- 1) Nonstorage capability. The resource defines the power injection to the grid. It can only be curtailed.
- 2) Limited storage of the converted energy. Example: thermal energy in solar thermal power plants can be stored.
- 3) Storage of primary energy – Low capacity
- 4) Storage of primary energy – Medium capacity
- 5) Storage of primary energy – High capacity
- **Dispatchability**: Capability of an electricity generation technology to provide power based on the operation setpoint.^[^ ^77^ ^]^ A source with full dispatchability implies being able to always reach the power setpoint regardless of the situation, while a plant with zero dispatchability can never provide the setpoint requested. Level definitions:
- 6) The primary energy availability permanently constrains the power output capability.
- 7) The primary energy availability constraints the power output capability, but the power can exceed the threshold temporarily (short time‐seconds)
- 8) The primary energy availability influences the power output capability. However, the power output can be increased by means of a secondary (inherent storage) energy source.
- 9) The primary energy availability is sufficient not to constrain the output power.
- 10)  The primary energy availability does not constrain the power output capability and it is possible to reverse the power plant to produce primary energy from the surplus of electricity in the network (bidirectional capability).
- **Response time**: The time elapsed between the acknowledgement of a new power reference and its successful tracking.
- **Inherent storage time**: The total amount of time that an electricity generation technology can provide electricity at full capacity by means of its inherent energy storage.^[^ ^12^ ^]^
- **CO_2_ emissions**: Amount of CO_2_ grams per kWh produced by an electricity generation technology considering its lifecycle footprint.
- **Levelized cost of electricity (LCOE)**: Average revenue per unit of electricity generated that would be required to recover the costs of building and to operate a generating plant during an assumed financial life and duty cycle.^[^ ^18^ ^]^
- **Capital expenditure (CAPEX)**: Funds used to acquire, upgrade, and maintain physical assets such as property, plants, buildings, technology, or equipment.
- **Operational expenditure (OPEX)**: Expenses related to the production of goods and services.

**Tables** **1** and **2** show all previous characteristics for the different generation technologies. These aspects determine each technology's role in the electric power system. PV and wind technologies present faster response times (from milliseconds to a few seconds) than those based on synchronous generators. However, PV and wind inherent storage time are zero, whereas other technologies offer this characteristic, which ranges from hours to months (conventional plants). Although these characteristics are not directly used in the optimization described in Section 4, they are reflected in the data used in the optimization, e.g. daily and monthly generation.

**Table 1:** Technical characteristics of the different generation technologies considered

	Response time	Inherent storage time	Controllability ^(1‐5)^	Dispatchability ^(1‐5)^	Generation technology
PV	100 ms–3 s ^(6‐7)^	0	1	1	PE
ST	15 min–4 h^a)^,^(8)^	0–24 hours ^(9)^	2	3	SG
W	0.5 ms–1 s ^(10)^	Few seconds	1	2	SG/IG+PE
HYD	2–5 min ^(11)^	4 h–16 h ^(12)^	3	4	SG
BIO	10 min–6 h^b)^,^(8)^	Weeks	4	4	SG
CF‐TPS	80 min–8 h ^(13)^	Months	5	4	SG
CC‐TPS	5 min–3 h ^(13)^	Months	5	4	SG
N‐TPS	∼24 h ^(8)^	18–24 months	5	4	SG
PS‐HPP	15–30 s ^(11)^	Minutes–years ^(12)^	3	5	SG
GEO	30 s–2 min	inf	5	4	SG

^a)^ Ramping rate: 6% of full load/min. Hot start‐up time: 2.5 h

^b)^ Ramping rate: 8% of full load/min. Hot start‐up time: 3 h.

(1)^[^ ^28^ ^]^, (2) (European Commission, 2012), (3)^[^ ^74^ ^]^, (4)^[^ ^16^ ^]^, (5)^[^ ^35^ ^]^, (6^[^ ^9^ ^]^, (7)^[^ ^5^ ^]^, (8)^[^ ^32^ ^]^, (9)^[^ ^60^ ^]^, (10)^[^ ^1^ ^]^, (11)^[^ ^29^ ^]^, (12)^[^ ^23^ ^]^, (13)^[^ ^42^ ^]^

Legend: PV: solar photovoltaic; ST: solar thermal; W: wind; HYD: hydropower; BIO: biomass; CF‐TPS: coal‐fired thermal power station; CC‐TPS: combined‐cycle thermal power station; N‐TPS: nuclear thermal power station; PS‐HPP: pumped‐storage hydropower plant; GEO: geothermal; PE: power electronics; SG: synchronous generator; SG/IG+PE: synchronous generator with power electronics or induction generator with power electronics.

**Table 2:** Costs and emissions of the different generation technologies considered

	LCOE [$ kWh^−1^]	CAPEX^(1)^ [$ kW^−1^]	OPEX^(1)^ [$ kW^−1^]	Fuel cost	CO_2_ Emissions ^(2),(3)^ [g‐eq kWh^−1^]
PV	0.029–0.190 ^(4,9)^	1313	15.25	0	18–180
ST	0.126‐0.156 ^(4)^	7221	85.40	0	9–63
W	0.026–0.099 (onshore), 0.086–0.162 (offshore) ^(4,9)^	1265–4375	26.34–110	0	8–40
HYD	0.02–0.05 ^(5,9)^	2500–15 993^(10)^	29.86	0	2–200
BIO	0.057–0.097 ^(5,9)^	4097	27.47	21.8–38.7 € MWh^−1(11)^	50–400
CF‐TPS	0.065–0.159 ^(4)^	3676–5876	40.58–59.54	10.33–50.31 € MWh^−1 (6,11)^	850–1125
CC‐TPS	0.044–0.073 ^(4)^	958–2481	12.20–27.60	22.55–32.25 € MWh^−1 (7,11)^	450–525
N‐TPS	0.129–0.198 ^(4)^	6041–6191	95.00–125.72	3‐5 € MWh^−1 (8)^	15–30
PS‐HPP	0.0473 ^(5)^	5316	29.86	0	2–200
GEO	0.05–0.101 ^(4,9)^	2521	129.70	0	50

(1)^[^ ^19^ ^]^, (2)^[^ ^76^ ^]^, (3)^[^ ^41^ ^]^, (4)^[^ ^48^ ^]^, (5)^[^ ^44^ ^]^, (6)^[^ ^20^ ^]^, (7)^[^ ^21^ ^]^, (8)^[^ ^70^ ^]^, (9)^[^ ^45^ ^]^, (10)^[^ ^59^ ^]^, (11)^[^ ^35^ ^]^.

## 3 Scenarios Generation

Most of the widely used power system benchmark systems does not consider RES in the generation, such as the IEEE and CIGRE test cases.^[^ ^64^ ^]^ Moreover, they lack to provide a clear rationale for the resource type and location in the grid. **Table** **3** summarizes common test systems.As the power systems are evolving to support a massive integration of RES, both the resources and the grid play a key role when defining modern power system scenarios. Therefore, in this section, several scenarios are defined in order to illustrate different power systems with different characteristics, such as the grid configuration or the combination of RES technologies in the system. The classification criteria are defined as follows:

- Three main grid configurations:
- Type I: Isolated
- Type II: Synchronously interconnected (AC)
- Type III: Nonsynchronously interconnected (DC) (i.e., isolated systems with only DC interconnection/s)
- Combination of different RES technologies:
- Different portions of RES in the system
- Controllable and noncontrollable technologies
- Consider power electronics in the generation plants
- In terms of grid layout, only transmission or transmission plus distribution
- Optionally, nonelectrochemical storage can be included

**Table 3:** Overview of standard power system benchmarks

Test system	AC/DC	Number of buses/nodes	Number of machines/converters	RES considered
WSCC 9 **^(1)^**	AC	9	3	×
IEEE 14 **^(1)^**	AC	14	5	×
IEEE 30 **^(1)^**	AC	30	6	×
IEEE 39 **^(1)^**	AC	39	10	×
IEEE 118 **^(1)^**	AC	118	54	×
IEEE 300 **^(1)^**	AC	300	69	×
CIGRE B4 DC **^(2)^**	DC	26	13	×
CIGRE HV **^(3)^**	AC	13	4	×
CIGRE MV **^(3)^**	AC	14	Utility	×
CIGRE LV **^(3)^**	AC	31	Utility	×
WECC 240 **^(4)^**	AC	240	146	√

(1)^[^ ^64^ ^]^, (2)^[^ ^79^ ^]^, (3)^[^ ^10^ ^]^, (4)^[^ ^80^ ^]^.

**Figure 11:**
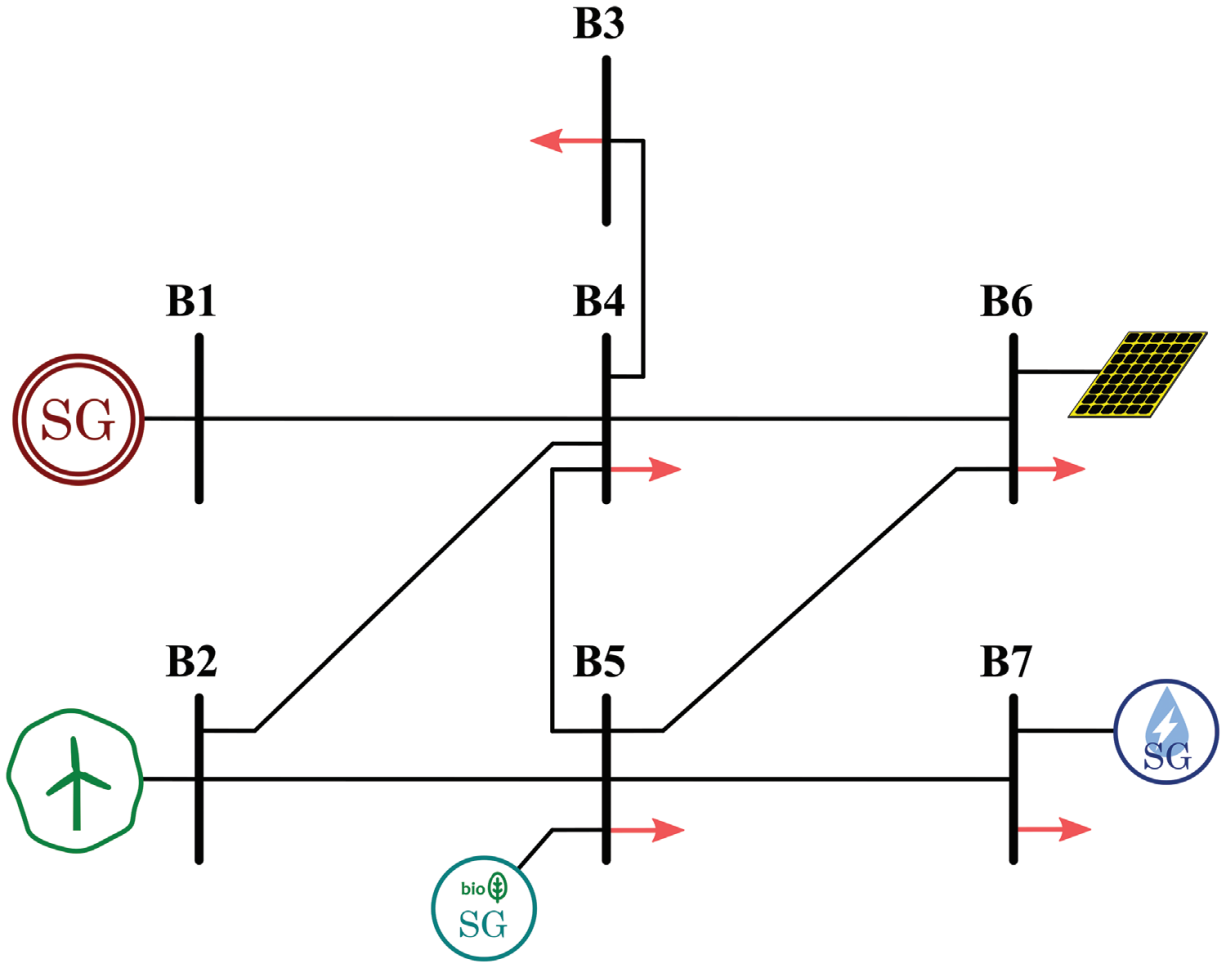
Selected scenario 1: Type I.

**Figure 12:**
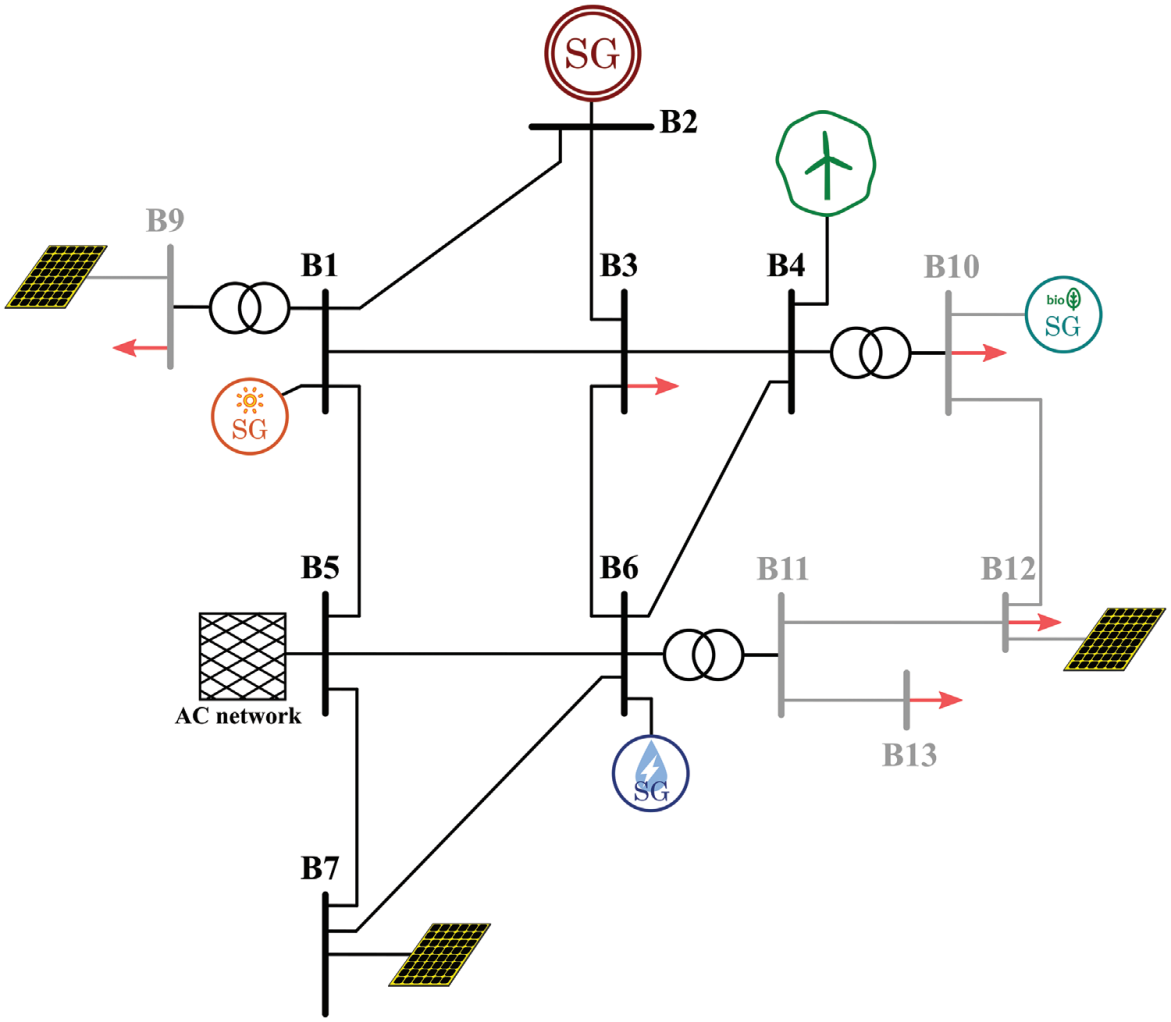
Selected scenario 2: AC interconnected (type II, southern Europe).

**Figure 13:**
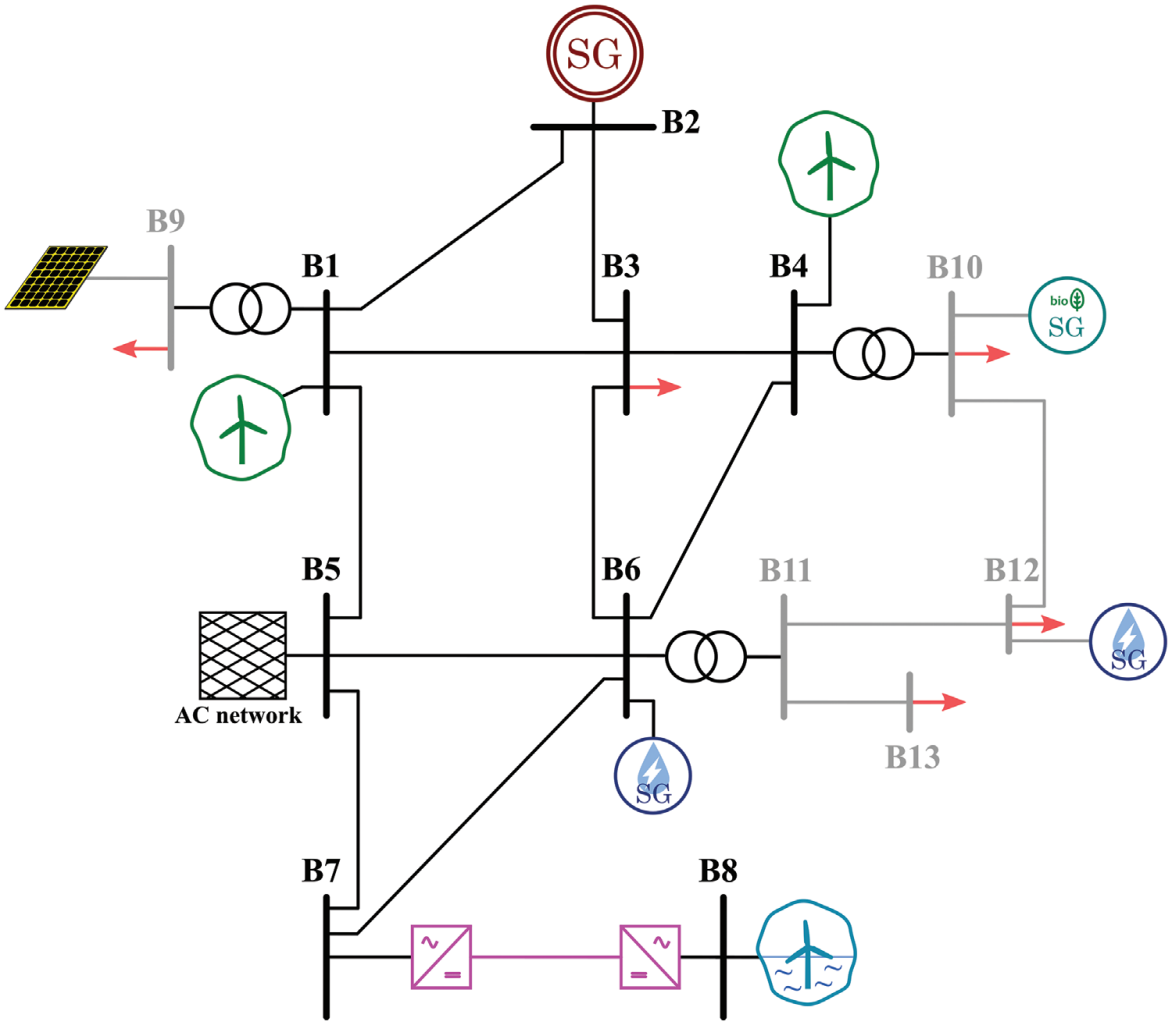
Selected scenario 3: AC interconnected (type II, northern Europe).

**Figure 14:**
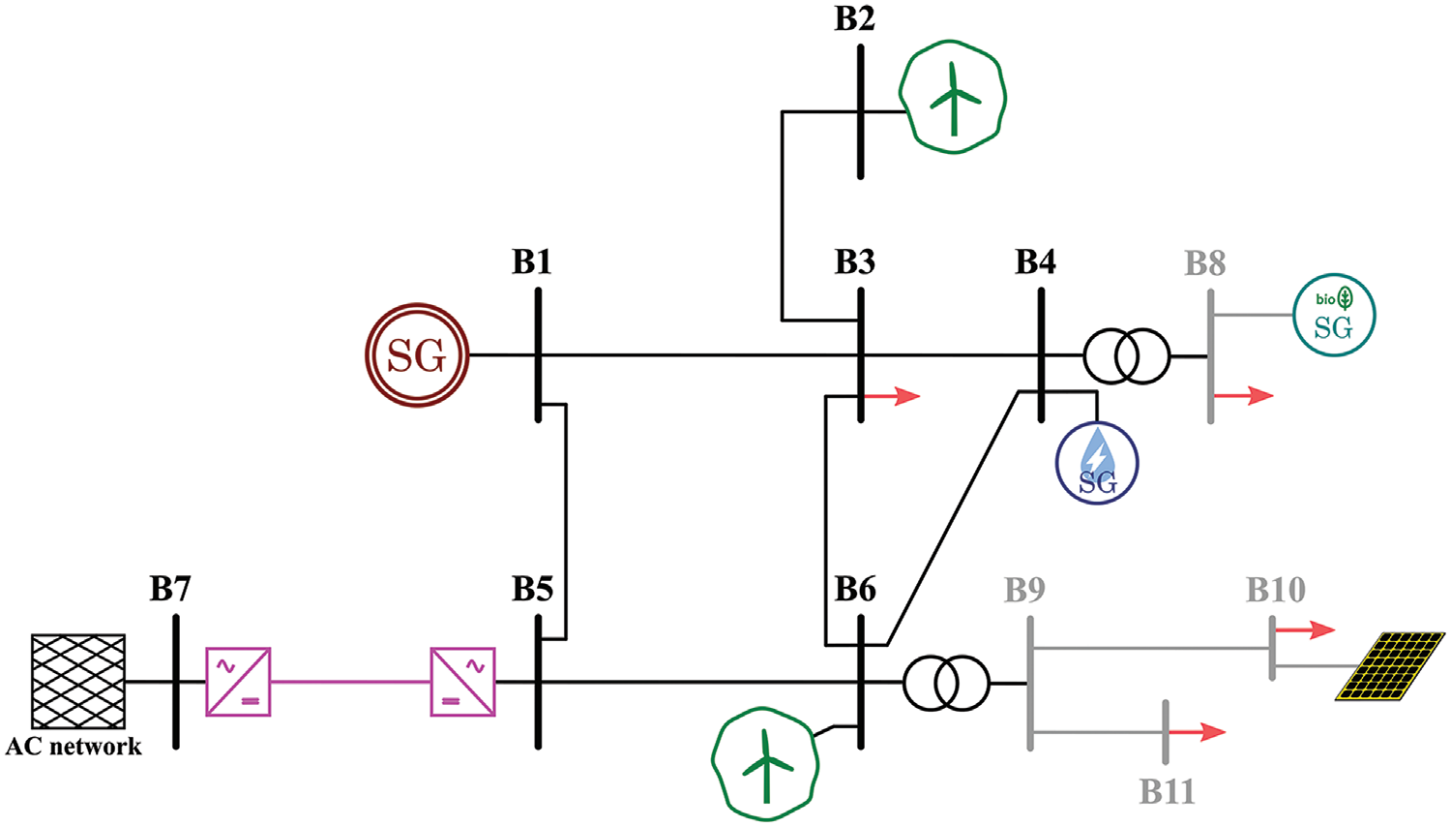
Selected scenario 4: DC interconnected (type III).

Based on the already existing scenarios in Europe, four realistic scenarios have been built as examples of power systems based on the different previously mentioned characteristics:

- Type I: Island scenarios are generally smaller and more straightforward than continental ones. Therefore, a smaller number of buses (in this case, seven) and a single voltage level is considered for this case (**Figure** **11**).
- Type II: The majority of scenarios is AC interconnected systems, and they are typically bigger and highly meshed. Consequently, a higher number of buses (in this case, thirteen) and different voltage levels (i.e., transmission and distribution) are considered. Adding distribution systems reflects better modern scenarios with a significant presence of distributed generation. Moreover, two distinct versions of this type of scenario are considered. One corresponds to a typical southern Europe scenario (**Figure** **12**), whereas the other corresponds to a typical northern Europe scenario (**Figure** **13**), including HVDC interconnected offshore wind.
- Type III: HVDC interconnected scenarios without AC interconnections typically correspond to bigger islands. Therefore, the considered grid layout is slightly more complex, with a higher number of buses than Type I. Additionally, different voltage levels are considered in this case (**Figure** **14**).

These scenarios are still preliminary since the power ratings of the transmission lines and the generation units are not defined. Based on these layouts, the algorithm described in Section 4 assigns an optimal rating to each element in the system, considering several inputs and restrictions.

## 4 Methodology for Sizing the Scenarios

In this section, a methodology to size the renewable generation technologies for realistic scenarios is described. The objective is to quantify the elements included in the previous scenarios, such as the rated capacity of the power plants or the capacity of the transmission lines. The sizing methodology is based on a generation cost optimization, considering the European or local policies regarding the objectives of renewable generation. The grid restrictions are not considered in this algorithm. The optimization quantifies the renewable generation that should be installed to fulfil the minimum share of renewable generation while minimizing the total generation cost.

Based on the characteristics and system topologies mentioned above, a large number of scenarios can be generated. Then, the optimization algorithm was applied only to Scenario 1 in Figure 11 and Scenario 3 in Figure 13 to exemplify potential results that could be obtained using this methodology. New scenarios can be easily generated by modifying the initial ones.

### 4.1 Generation Cost Optimization

**Figure 15:**
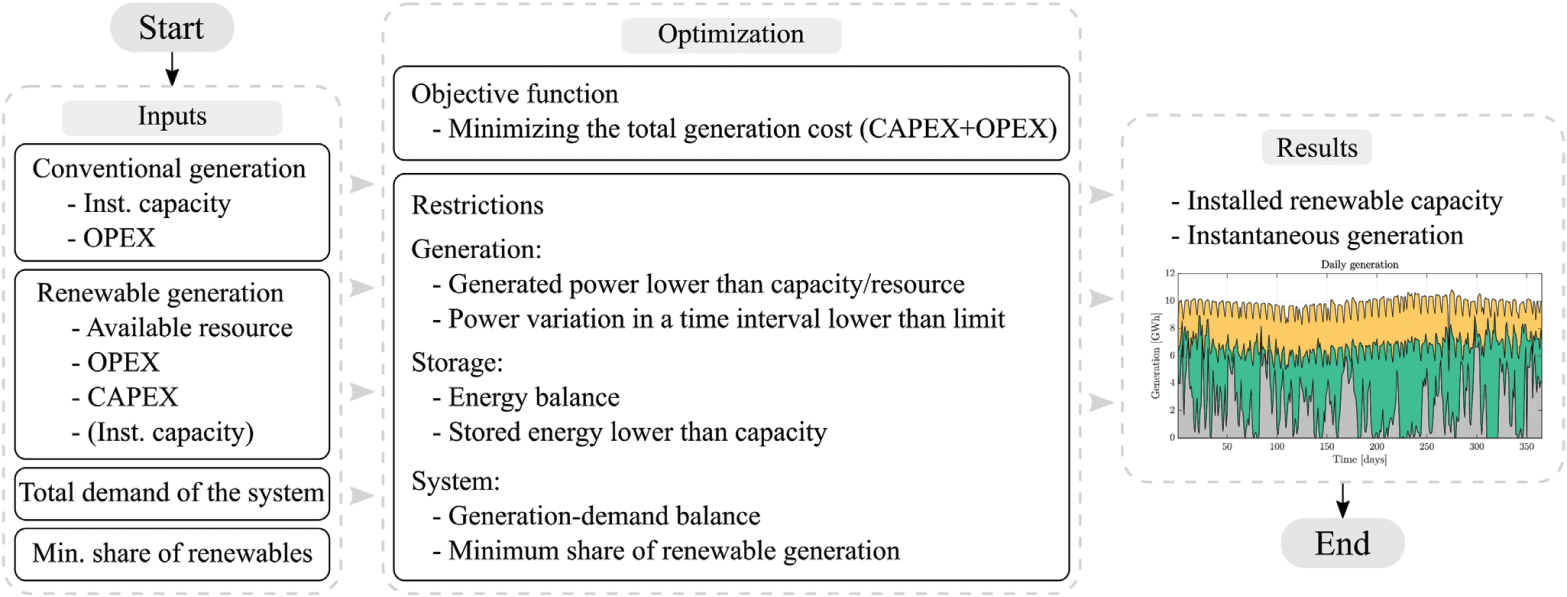
Overview of the optimization algorithm.

The optimization algorithm has been developed in Python in order to obtain the renewable capacity that minimizes the generation costs. A flowchart of this algorithm is shown in **Figure** **15**. Several inputs are required in order to define the power plants and system characteristics:

- Conventional generation: It is assumed to be already installed in the system, so CAPEX is not considered. Then, the inputs required for the conventional thermal power plants are the installed capacity and the OPEX.
- Renewable generation: Both CAPEX and OPEX are considered as inputs. In addition, the availability of resources, i.e., irradiation or wind speed, is also required. If a renewable power plant has already been built, the CAPEX is no longer needed, but the installed capacity is instead.
- System: The total demand at each time interval and the minimum share of renewable generation.

The previous inputs are defined explicitly for each conventional and renewable generation model.

### 4.2 Modeling of the System Elements

Conventional and renewable generation models have been implemented in Python to represent the particular characteristics of every energy resource. Four models have been considered: conventional power plants, renewable power plants without storage (PV and wind), solar thermal power plants, and pumped‐storage hydropower plants. System restrictions are also included in the model.

#### 4.2.1 Conventional Power Plants

Conventional power plants, e.g. coal or gas‐based power plants, are represented by the following restriction:

1) Maximum power generation: the instantaneous power generation (*G*_Ci_) must be lower or equal to the installed capacity of the power plant.
(1) $$\begin{array}{c} x_{GCi,t}\leq G_{Ci}\forall i,\forall t \end{array}$$

where *i* denotes each of the *C*_i_ conventional power plants, *x*_GCi,t_ is the instantaneous generation of the conventional power plant *C*_i_ at the time *t*, and *G*_Ci_ is the installed capacity of the conventional power plant *C*_i_.

#### 4.2.2 Renewable Generation Without Storage (PV and Wind)

The PV, wind power plants, and conventional power plants model share the same restriction. However, in this case, the maximum generation will depend on the availability of the resource:

1) Maximum power generation:
(2) $$\begin{array}{c} x_{GRj,t}\leq G_{Rj}\cdot C_{Rj}\forall j,\forall t \end{array}$$

where *j* refers to each of the *R*_j_ renewable power plants, *x*_GRj,t_ is the instantaneous generation of the renewable power plant *R*_j_ at the time *t*, *G*_Rj_ is the installed capacity of the renewable power plant *R*_j_ and *C*_Rj_ are the available resource expressed in pu. Solar and wind resources were obtained from.^[^ ^69^ ^]^

#### 4.2.3 Pumped‐Storage Hydropower Plants

The hydropower plants have been considered pumped‐storage plants without external contributions of water. Then, the energy stored only depends on the pumping and turbine power balance. The PS‐HPPs have been modeled as follows:

1) Maximum power generation/consumption: The same rated power has been considered for pumping and turbine power.
(3) $$\begin{array}{c} x_{GHk,t}\leq G_{Hk}\forall k,\forall t \end{array}$$
(4) $$\begin{array}{c} x_{PHk,t}\leq G_{Hk}\forall k,\forall t \end{array}$$

where *k* refers to each of the *H*_k_ hydropower plants, *x*_GHk,t_ and *x*_PHk,t_ are the instantaneous generation and pumping power of the hydropower plant *H*_k_ at the time *t*, respectively, and *G*_Hk_ is the installed capacity of the hydropower plant *H*_k_.

2) Energy balance of the storage system:
(5) $$\begin{array}{c} x_{Sk,t+1}=x_{Sk,t}+x_{PHk,t}\cdot \eta_{P}-\frac{x_{GHk,t}}{\eta_{G}}\forall k,\forall t\varepsilon \text{​}\left[1,T-1\right] \end{array}$$

where *x*_Sk,t_ is the energy stored in the hydropower plant *H*_k_ at the time *t* and η_P_ and η_T_ are the pumping and turbine efficiencies, respectively. *x*_GHk,t_ and *x*_PHk,t_ are the same variables used in (3) and (4).

3) Maximum energy storage: The maximum energy storage is defined by the capacity of the upper reservoir of the hydropower plant.
(6) $$\begin{array}{c} x_{Sk,t}\leq S_{Hk}\forall k,\forall t \end{array}$$

where *S*_Hk_ is the storage capacity of the hydropower plant *H*_k_ and *x*_Sk,t_ was defined in (5).

#### 4.2.4 Solar Thermal Power Plants

The solar thermal power generation represented in this study is based on parabolic troughs, as it is the main technology used in the solar thermal power plants.^[^ ^73^ ^]^ These power plants are usually equipped with thermal storage systems, which are sized to have the capability to maintain rated power for several hours. Then, the solar thermal generation model includes the thermal power absorbed by the parabolic troughs, the thermal storage, and the electric generation:

1) Maximum thermal power absorbed by the solar field: The thermal power absorbed by the parabolic troughs highly depends on the solar irradiation and the angle of incidence:^[^ ^6^ ^]^
(7) $$\begin{array}{c} x_{TSl,t}\leq i_{Sl,t}\cdot r_{l}\cdot G_{Sl}\cdot \eta_{O,l}\cdot \eta_{Ef}\cdot K\left(\theta \right)\forall l,\forall t \end{array}$$

where *l* refers to each of the *S*_l_ solar thermal power plants, *x*_TSl,t_ is the thermal power absorbed by the *S*_l_ solar thermal power plant at time *t*, *i*_Sl,t_ is the solar irradiation in kW m^−2^, *r*_l_ is a ratio which relates the solar field surface needed to generate 1 kWe (electric power kW),^[^ ^6^ ^]^ *G*_Sl_ is the rated electrical power of the plant, η_O,l_ is the peak optical efficiency, η_Ef_ is an efficiency factor which considers other losses, such as thermal, cleanliness, or tracking losses, and *K*(θ) is a factor obtained from the angle of incidence. *K*(θ) can be calculated as:^[^ ^30^ ^]^

(8) $$\begin{array}{c} K\left(\theta \right)=1-\frac{7\cdot 10^{-4}\cdot \theta +36\cdot 10^{-6}\cdot \theta^{2}}{\cos\theta } \end{array}$$

where θ is the angle of incidence, defined for a specific location and time.^[^ ^57^ ^]^

2) The Maximum electric power is restricted by the rated power:
(9) $$\begin{array}{c} x_{GSl,t}\leq G_{Sl}\forall l,\forall t \end{array}$$

where *x*_GSl,t_ is the electric power generation of the solar thermal power plant *S*_l_ at the time *t* and *G*_Sl_ was defined in (7).

3) Energy balance of the thermal storage system: the thermal energy stored varies based on the thermal and electric powers as:
(10) $$\begin{array}{c} x_{Sl,t+1}=x_{Sl,t}+x_{TSl,t}-\frac{x_{GSl,t}}{\eta_{th,l}}\forall l,\forall t\varepsilon \left[1,T-1\right] \end{array}$$

where *x*_Sl,t_ is the energy stored in the solar thermal power plant *S*_l_ at the time *t* and η_th_ is the thermoelectric efficiency of the thermal power plant, *x*_TSl,t_ was defined in (7) and *x*_GSl,t_ is defined in (9). An ideal storage system has been assumed, so storage losses are not considered.

4) Maximum thermal energy storage: the maximum thermal energy is defined by the capacity of the storage tank:
(11) $$\begin{array}{c} x_{Sl,t}\leq S_{Sl}\forall l,\forall t \end{array}$$

where *S*_Sl_ is the storage capacity of the solar thermal power plant *S*_l_, and *x*_Sl,t_ is defined in (10).

#### 4.2.5 Power System

The power system has been modeled using an aggregated representation, which only considers the total system demand. The grid equations are not included in the model. Then, two restrictions are implemented:

1) Generation‐demand balance: The power generation must meet the total system demand for every time interval.
(12) $$\begin{array}{c} \sum_{i\text{​}=\text{​}1}^{I}x_{GCi,t}+\sum_{j\text{​}=\text{​}1}^{J}x_{GRj,t}+\sum_{k\text{​}=\text{​}1}^{K}x_{GHk,t}+\sum_{l\text{​}=\text{​}1}^{L}x_{GSl,t}=D_{t}+\sum_{k\text{​}=\text{​}1}^{K}x_{PHk,t} \end{array}$$

where *I* is the number of conventional power plants, *J* is the number of PV and wind power plants, *K* is the number of pumping hydropower plants, and *L* is the number of solar thermal power plants. *D*_t_ is the system demand at the time *t*. The variables *x*_GCi,t_, *x*_GRj,t_, *x*_GHk,t_, *x*_PHk,t_, and *x*_*GSl*,*t*_ were defined in (1), (2), (3), (4) and (9), respectively.

2) The minimum contribution of renewable generation during a year:
(13) $$\begin{array}{c} \sum_{t\text{​}=\text{​}1}^{T}\left(\sum_{j\text{​}=\text{​}1}^{J}x_{GRj,t}+\sum_{l\text{​}=\text{​}1}^{L}x_{GSl,t}\right)>=\alpha \sum_{t\text{​}=\text{​}1}^{T}D_{t} \end{array}$$

where α is the minimum share of renewable generation expressed in per unit and *T* is the entire time period (one year in this study). Pumping hydro generation is not included as renewable generation, as its net energy contribution is null or even negative if the pumping and turbine efficiencies are considered.

### 4.3 Optimization Problem

The optimization algorithm provides the generation mix that minimizes the cost for the system. Then, the objective function *f*_obj_(*x*) of the optimization function can be defined as:

(14) $$\begin{array}{c} \begin{array}{c} f_{\text{obj}}\left(x\right)={\text{OPEX}}_{\text{Conv}}\left(x\right)+{\text{CAPEX}}_{\text{Ren}}\left(x\right)+{\text{OPEX}}_{\text{Ren}}\left(x\right)\\ \text{subject}\;\text{to}\;h_{m}\left(x\right)=0,m\varepsilon \left[1,M\right]g_{n}\left(x\right)\leq 0,n\varepsilon \left[1,N\right] \end{array} \end{array}$$

where *x* is the variable vector, *OPEX*_Conv_(*x*) is the operation cost of the conventional power plants, *CAPEX*_Ren_(*x*) is the capital cost of the renewable generation that must be installed, *OPEX*_Ren_(*x*) is the operation cost of the renewable generation, *h*_m_(*x*) is the *m*‐th equality constraint and *g*_n_(*x*) is the *n*‐th inequality constraint. The renewable generation category in this optimization also includes HVDC interconnected generation. This aggregation approach reduces the number of categories and facilitates the optimization and analysis.

The variable vector *x* includes the instantaneous generation for every time interval for all the generation types considered, as well as the installed capacity of the renewable generation. Then, the variable vector can be defined as:

(15) $$\begin{array}{c} x=\left[x_{C},C_{R},x_{R},C_{H},x_{H},C_{S},x_{S}\right] \end{array}$$

where

(16) $$\begin{array}{c} x_{C}=\left[x_{GCi,1},\text{…},x_{GCi,T}\right]\forall i \end{array}$$

(17) $$\begin{array}{c} C_{R}=\left[C_{R1},\text{…},C_{RJ}\right] \end{array}$$

(18) $$\begin{array}{c} x_{R}=\left[x_{GRj,1},\text{…},x_{GRj,T}\right]\forall j \end{array}$$

(19) $$\begin{array}{c} C_{H}=\left[C_{H1},\text{…},C_{HK}\right] \end{array}$$

(20) $$\begin{array}{c} x_{H}=\left[x_{GHk,1},\text{…},x_{GHk,T},x_{PHk,1},\text{…},x_{PHk,T}\right]\forall k \end{array}$$

(21) $$\begin{array}{c} C_{S}=\left[C_{S1},\text{…},C_{SL}\right] \end{array}$$

(22) $$\begin{array}{c} x_{S}=\left[x_{GSl,1},\text{…},x_{GSl,T}\right]\forall l \end{array}$$

The cost functions are defined as:

(23) $$\begin{array}{c} {\text{OPEX}}_{\text{Conv}}\left(x\right)=\sum_{t=1}^{T}\left(\sum_{i=1}^{I}x_{GCi,t}\cdot {\text{OPEX}}_{Ci}\right) \end{array}$$

(24) $$\begin{array}{c} {\text{CAPEX}}_{\text{Ren}}\left(x\right)=\sum_{j=1}^{J}C_{Rj}\cdot {\text{CAPEX}}_{Rj}+\sum_{k\text{​}=\text{​}1}^{k}C_{Hk}\cdot {\text{CAPEX}}_{Hk}+\sum_{l\text{​}=\text{​}1}^{L}C_{\text{Sl}}\cdot {\text{CAPEX}}_{\text{Sl}} \end{array}$$

(25) $$\begin{array}{c} {\text{OPEX}}_{\text{Ren}}\left(x\right)=\sum_{t=1}^{T}\left(\sum_{j=1}^{J}x_{Rj}\cdot {\text{OPEX}}_{Rj}+\sum_{k=1}^{K}x_{Hk}\cdot {\text{OPEX}}_{Hk}+\sum_{l=1}^{L}x_{Sl}\cdot {\text{OPEX}}_{\text{Sl}}\right) \end{array}$$

Where the variables *x*_GCi,t_ and *C*_Rj_ are defined in (1) and (2), respectively, *x*_Rj_ is the *j*‐th value of the vector *x*_R_ in (18), *x*_Hk_ is the *k*‐th value of the vector *x*_H_ in (20) and *x*_Sl_ is the *l*‐th value of the vector *x*_Sl_ in (22). The equality and inequality constraints, *h*_m_(*x*) and *g*_n_(*x*), are based on the models of the different generation types described in Section 4.2.

### 4.4 Limitations of the Method

The proposed optimization method to size test grids considering future scenarios considered mainly the energetics, which is mainly related to the sources of power and the demand. In order to perform such optimization, several simplifications were performed, listed as follows:

- No electrical grid constraints were considered.
- The topology of the converters interfacing the RES in the wind and PV cases was not considered. Thus, no difference was considered between grid‐following and grid‐forming converter technologies.
- The grid stability (transient, small signal, harmonic stability) was not considered.
- The only storage considered was the one provided by pumped‐storage hydropower plants. Thus, no battery storage system or other storage system was considered.
- The demand was given by real data and was considered not flexible. Thus, no demand flexibility was considered in the optimization.
- The optimization was performed in a deterministic basis, meaning that the uncertainty of wind and solar generation was not considered. Instead, real data of one full year was used.

## 5 Application of the Methodology to the Selected Scenarios

The previous methodology has been applied to some of the realistic scenarios shown in Section 3. These scenarios have been studied in the context of the POSYTYF project.^[^ ^67^ ^]^ Two cases have been selected in order to validate the methodology and exemplify the sizing of such scenarios:

- Scenario 1: Type I – Isolated: Island
- Scenario 3: Type II – Synchronously interconnected (AC): northern Europe

### 5.1 Scenario 1: Type I – Island

Tenerife has been chosen as the reference location to obtain the system demand and solar and wind resources availability. The hourly power demand in 2019 varied from 300 to 550 MW approximately.^[^ ^68^ ^]^ It has been assumed that a 600‐MW coal‐fired generation is already installed. Regarding RES, two possible cases have been considered for this scenario:

- Case 1 – Without storage: only PV and wind are considered.
- Case 2 – With storage: in addition to PV and wind RES generation technologies, solar thermal generation with storage has been included in the system.

#### 5.1.1 Case 1 – Without Storage

**Figure 16:**
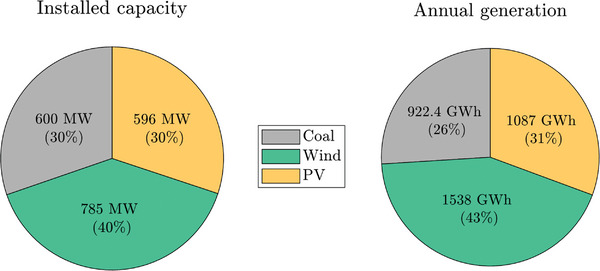
Installed capacity and annual generation mix for Case 1 and α = 74%.

First, the optimization has been executed to obtain the RES installed capacity for a 74% share of renewables (α), which is the target established by the Spanish government for 2030.^[^ ^74^ ^]^ To meet the previous requirement, the optimization algorithm has determined that wind and PV generation technologies should provide 43% and 31% of the total demand, respectively (see **Figure** **16**). Furthermore, the overall installed capacity of wind and PV should be 785 and 596 MW, respectively (Figure 16).

**Figure 17:**
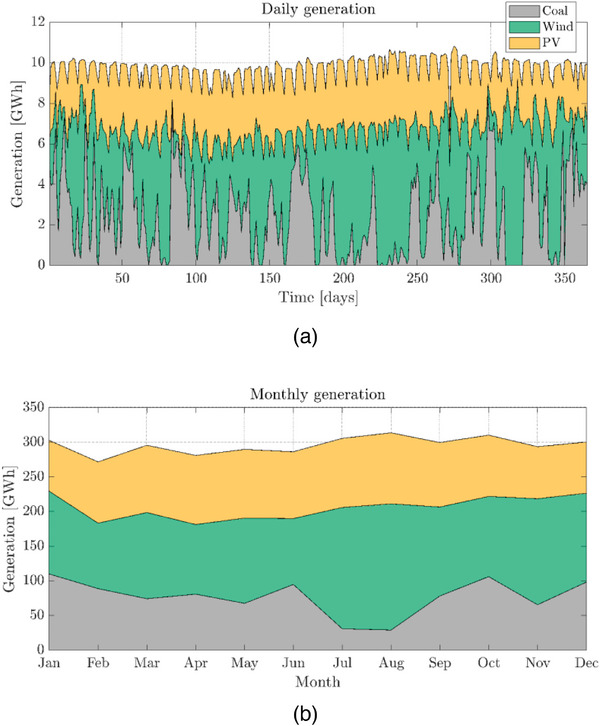
Generation mix for Case 1 in 2019: a) daily; b) monthly.

**Figure** **17** shows the generation mix for 2019 on a daily and monthly basis. It can be noticed that the PV generation pattern is very similar during the whole year. On the other hand, wind energy generation varies considerably and coal‐fired generation compensates the power variations. Regarding per month generation, July and August are the months with a higher contribution of wind energy, and January and October for a coal‐based generation.

**Figure 18:**
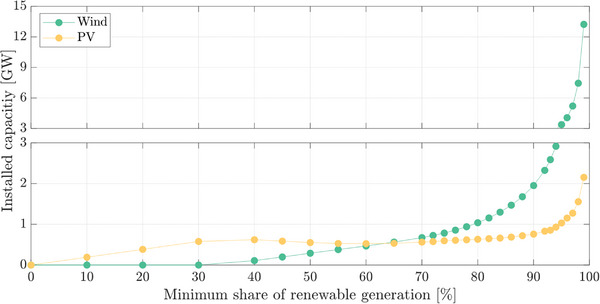
Installed capacity of wind and photovoltaic (PV) generation for Case 1

Additionally, the effect of the minimum share of renewables (α) on RES installed capacity has also been analyzed in **Figure** **18**. It can be observed that the exponential curve requires an extremely high installed capacity for α close to 100%. This is caused by the lack of solar and wind resources at some time intervals, i.e., low wind at night, forcing the algorithm to oversize them. For α = 100%, no solution is found as neither PV nor wind generation can supply the demand.

**Figure 19:**
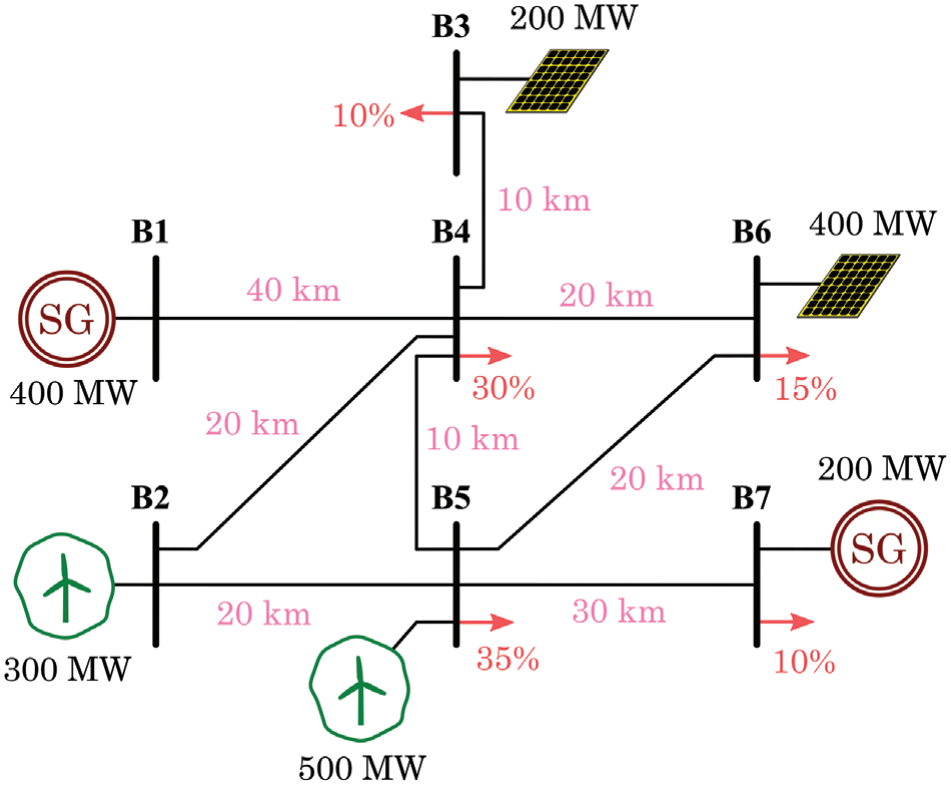
Scenario 1: Island without storage capability.

Based on the results obtained and the preliminary layout from Section 3 (Figure 11), the final scenario is depicted in **Figure** **19**. The scenario contains a total amount of seven transmission buses and no distribution. The obtained capacity for wind and PV is distributed in the different available buses, and two conventional generation units are considered. Different portions of the demand are distributed among the buses. The distances are relatively short, as this scenario represents a relatively small island. In such reduced systems, typical voltage levels used for the transmission lines are in the range of 66–220 kV.

#### 5.1.2 Case 2 – With Storage

To overcome the previous oversizing issue, storage elements can be introduced into the system. In this case, solar thermal power generation has been included in the optimization problem. A thermal storage system equivalent to 7.5 h of rated electric power has been assumed. The actual storage capacity will depend on the rated power of the solar thermal generation, which is also a variable of the optimization problem.

**Figure 20:**
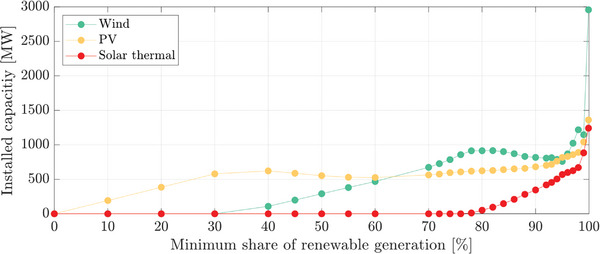
Installed capacity of wind, photovoltaic (PV), and solar thermal generation for Case 2.

**Figure 21:**
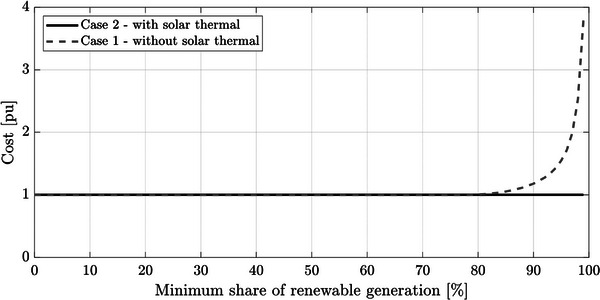
Total generation cost considering Case 2 as the base case.

Then, a similar analysis was carried out to study the renewable generation that must be installed for a different α, resulting in **Figure** **20**. Due to the higher cost of solar thermal generation, the algorithm does not include it in the solution until α ≥ 80%. Then, for α lower than 80%, Figure 20 is identical to Figure 18. For α above this value, the introduction of solar thermal generation avoids the oversizing of wind and PV generation. However, for α = 100%, the optimization provides a nonrealistic solution. More than 30 GW of wind energy is required to supply a system with a peak demand of around 550 MW (this singularity is not represented in Figure 20). For α = 99.9%, the obtained solution is still acceptable, although the required renewable capacity is considerably higher than those obtained for 99%. **Figure** **21** shows the generation cost comparison between Cases 1 and 2. The introduction of storage into the system allows a considerable cost reduction for α higher than 90%.

**Figure 22:**
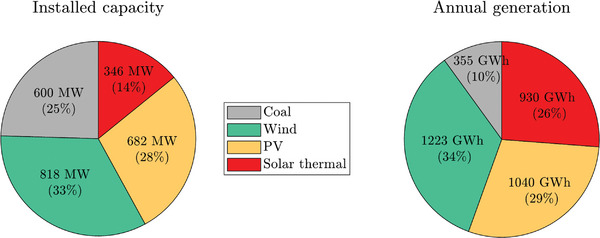
Installed capacity and annual generation mix for Case 2 and α = 90%.

A specific case when α = 90% has been selected to exemplify the system's performance when the solar thermal power plant is included. **Figure** **22** shows the installed capacity and the annual generation for every technology. It can be observed how renewables can supply 90% of the energy while their contribution to the installed capacity is only 75%. This is possible thanks to the storage system of the solar thermal power plant.

**Figure 23:**
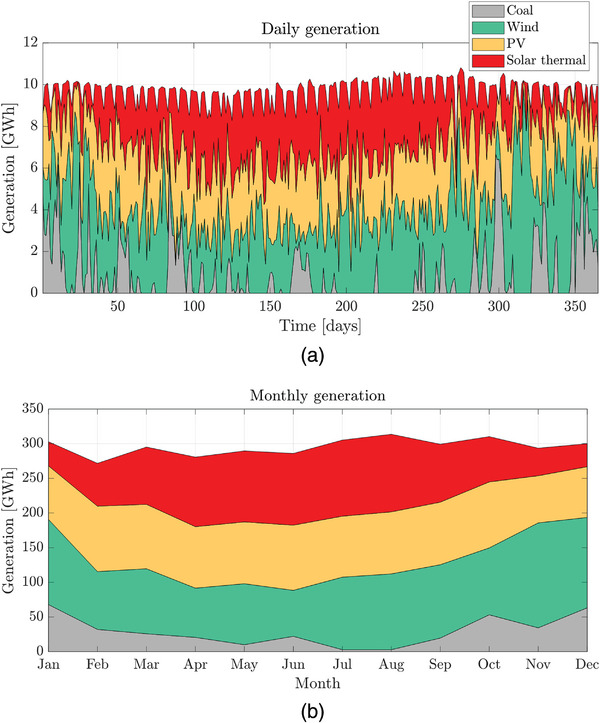
Generation mix for Case 2 in 2019:a) daily; b) monthly.

Additional specific results about daily and monthly generation are shown in **Figure** **23**. PV and solar thermal generation contributions are higher in summer, while coal has to compensate for the lack of solar resources in winter.

**Figure 24:**
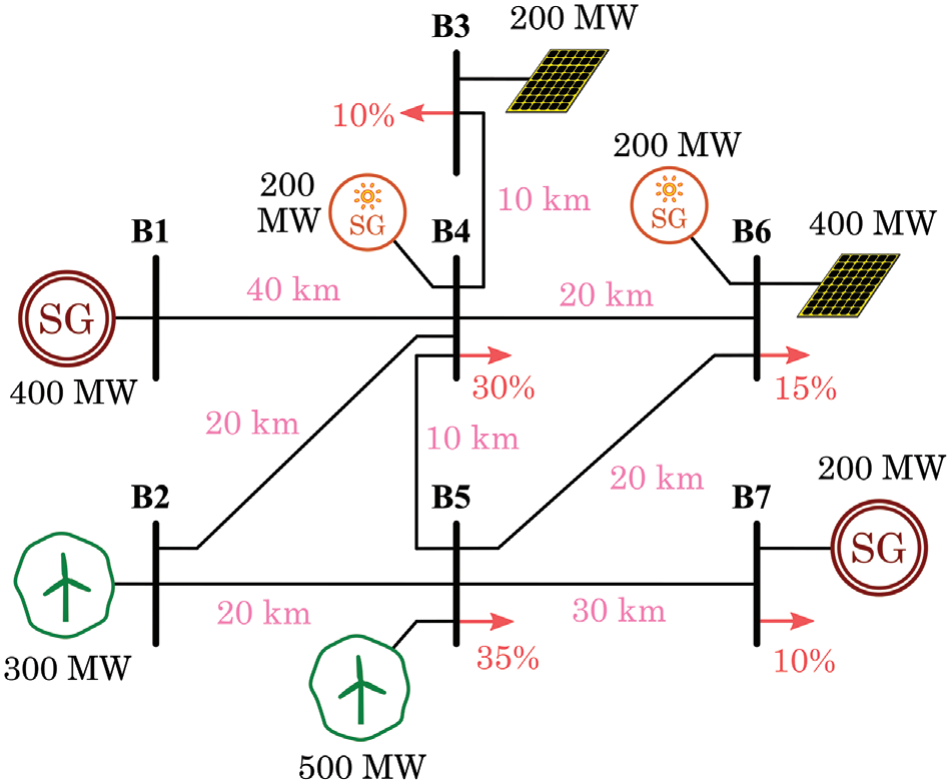
Scenario 2: Island without storage capability – Final proposed layout.

The same grid configuration is considered in this second case but including solar thermal (**Figure** **24**). The conventional, wind and PV obtained capacities are very similar to the case without solar thermal. Thus, the powers shown are the same as in the previous case. The additional solar thermal capacity is distributed equally in buses 4 and 6, in the same area where the PV is located.

#### 5.1.3 Scenario 3: Type II – Synchronously Interconnected (AC): Northern Europe

Scenario 3 corresponds to an AC interconnected system (Type II) located in northern Europe. This scenario has been specifically located in the Netherlands to extract the system demand and the solar and wind resources. The hourly consumption data of the Netherlands obtained^[^ ^61^ ^]^ has been scaled down to have a maximum instantaneous demand of 5 GW, leading to a minimum demand of 2.37 GW.

The generation technologies included in this scenario are coal, wind, PV, and PS‐HPP. Three cases have been considered to analyze the effect of water storage in the system:

- Case 1: No storage.
- Case 2: PS‐HPP generation with an installed capacity equal to 10% of the maximum demand.
- Case 3: PS‐HPP generation with an installed capacity equal to 20% of the maximum demand.

For Cases 2 and 3, PS‐HPP generation is assumed to be already installed, so the capital cost is not considered.

**Figure 25:**
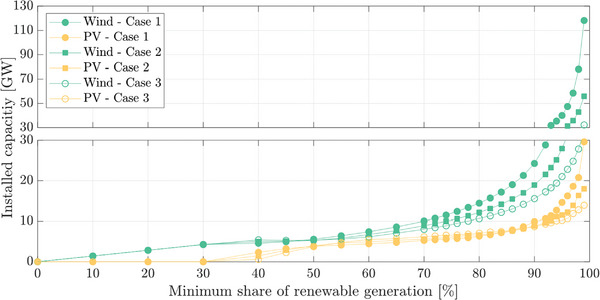
Installed capacity of wind and photovoltaic (PV).

**Figure** **25** shows the wind and PV capacity required based on the minimum renewable share. It is observed that PS‐HPP generation presence can help to reduce the amount of renewable generation considerably. For α = 90%, the wind capacity obtained for Case 1 is around 24 GW, while it is reduced to 19 GW for Case 2 and 15 GW for Case 3. So, a reduction of almost 10 GW of wind power can be achieved only by 1 GW of PS‐HPP generation.

**Figure 26:**
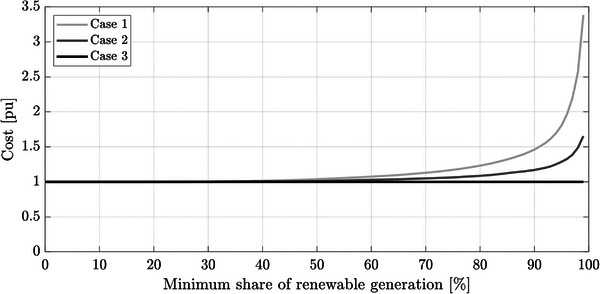
Total generation cost considering Case 3 as the base case.

This results in a reduction of the generation costs of the system, shown in **Figure** **26**. The costs have been normalized considering Case 3 as the base case. It can be observed that Case 3 provides a cost of around 50% and 20% lower than Case 1 and Case 2 when α = 90%. Higher cost reductions are achieved for higher values of α, as PS‐HPP generation avoids the installation of new wind and PV generation. However, it must be noted that the capital cost of PS‐HPP generation has not been considered. In that case, the cost reduction obtained would be lower.

**Figure 27:**
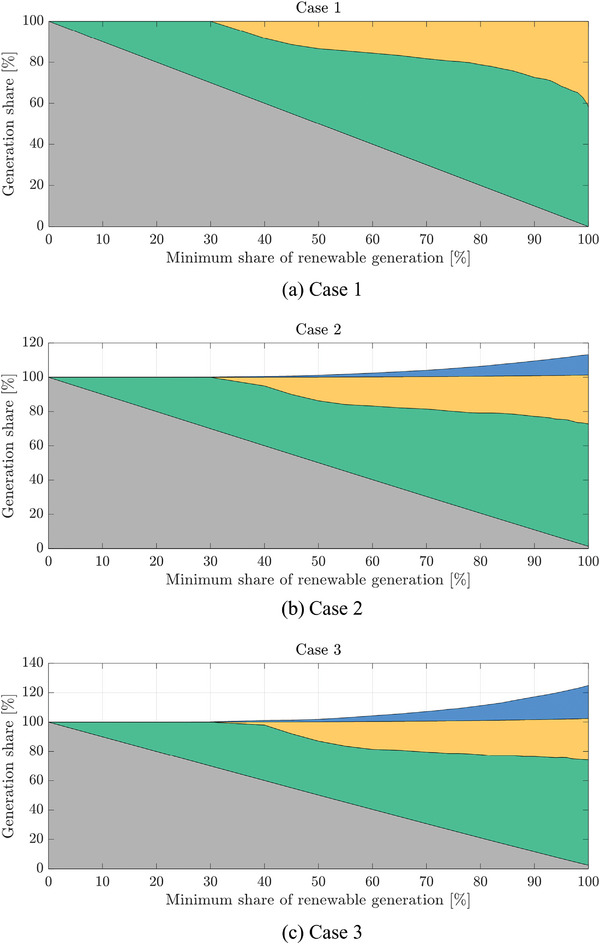
Generation share based on the minimum renewable required.

The generation mix with respect to α is depicted in **Figure** **27** for the three cases. Coal, wind, and PV generation share the demand, while hydrogeneration is always above 100%, as the net energy contribution of PS‐HPP is null. The use of hydro generation rises when α is increased, allowing storing energy and saving the installation of wind or PV. In Cases 2 and 3, the coal generation is different from zero when α is set to 100%. The restriction in (13) ensures that wind and PV generation is equal to the system demand. When PS‐HPP is included, this demand is increased due to the pumping consumption partially supplied by coal.

**Figure 28:**
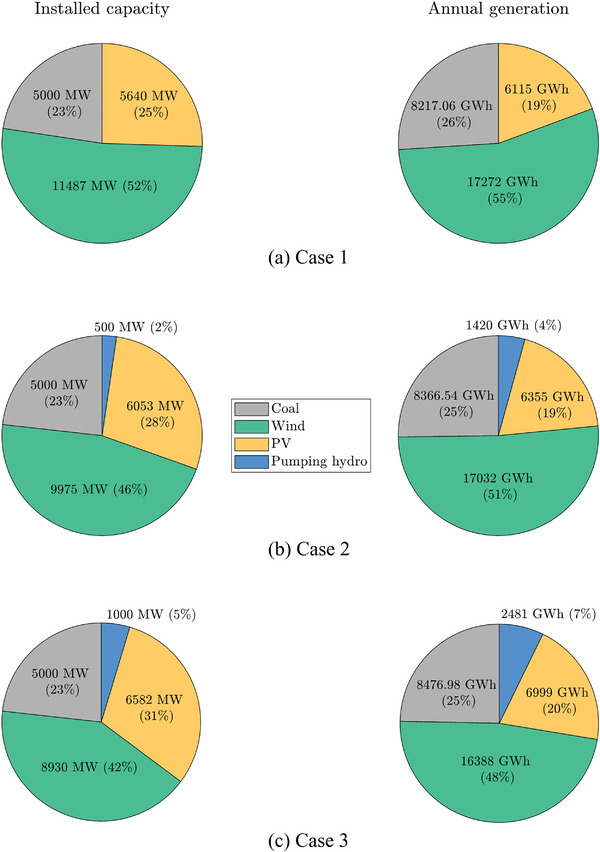
Installed capacity and annual generation mix for α = 74%.

Further analysis was carried out when α = 74%. **Figure** **28** shows the installed capacity and annual generation for all cases. The installation of PS‐HPP allows for supplying nearly the same amount of renewable generation reducing the installed capacity of wind and PV for Cases 2 and 3.

**Figure 29:**
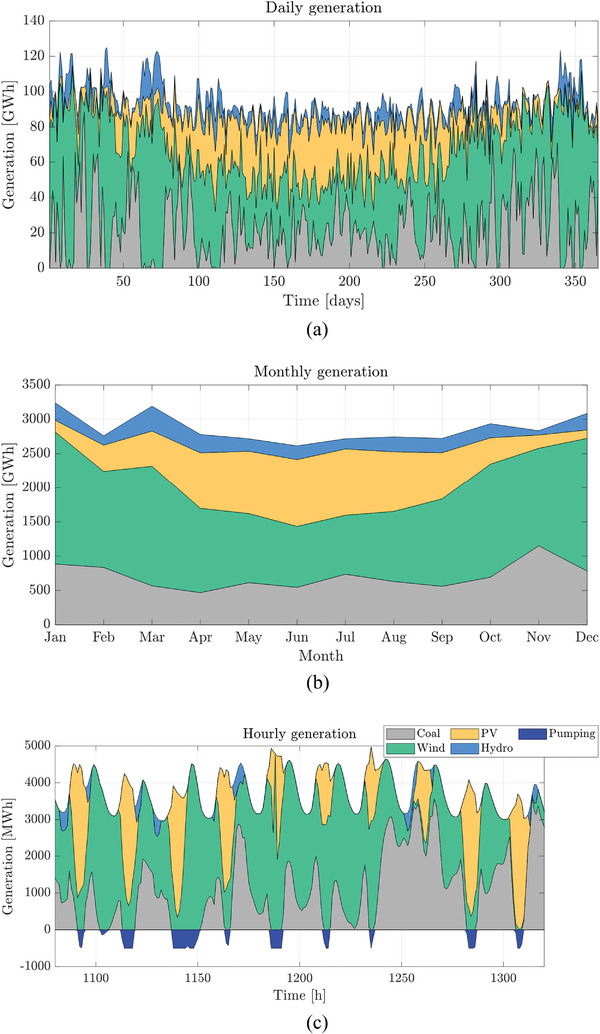
Daily, monthly, and hourly generation for Case 3 and α = 74%.

Specific daily, monthly and hourly results of Case 3 are shown in **Figure** **29**. In this case, the PV generation varies throughout the year, as Scenario 3 is located in a higher latitude than Scenario 1. Figure 29(c) shows how the pumping is used when there is a high renewable generation, helping to reduce the generation cost.

**Figure 30:**
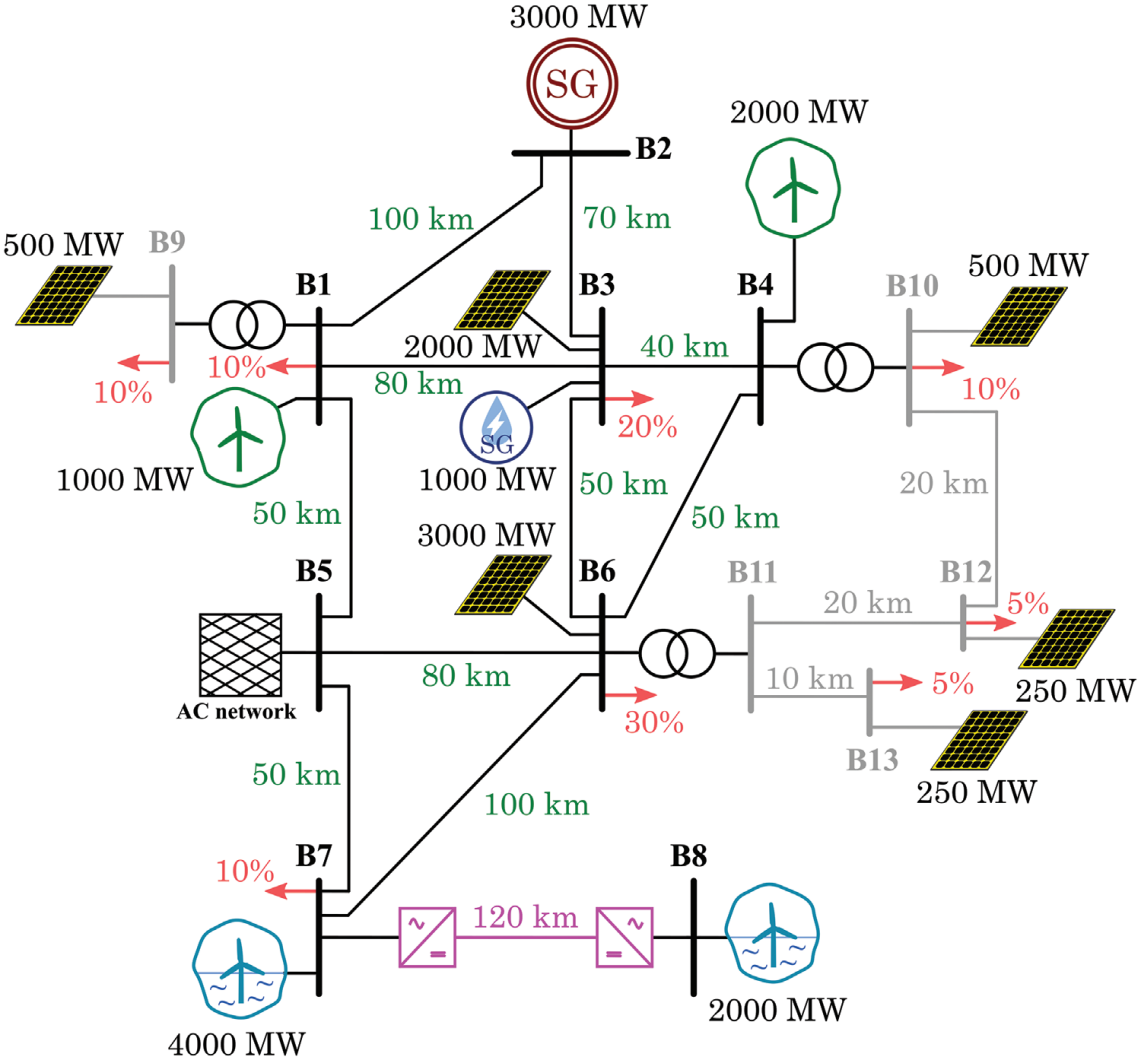
Scenario 3 – Final proposed layout.

Based on the results obtained and the preliminary layout from Section 3 (Figure 13), the final scenario is depicted in **Figure** **30**. The scenario contains a total amount of eight transmission buses and five distribution buses. The large size of the different power plants does not represent a single power plant but an aggregated equivalent of several ones. The voltage levels of the transmission lines could be, for instance, in the range of 220–400 kV, whereas in the distribution case, it could be 20–30 kV. An appropriate amount of offshore wind (both DC‐interconnected and AC‐interconnected) is considered, as this scenario is inspired in the north of Europe. Also, onshore wind, PV and PS‐HPP are considered, and a portion of conventional generation.

## 6 Conclusion

This overview has presented different possible scenarios that can be used for the analysis of the large integration of RES in Europe. The design of the scenarios has been done considering specific weather conditions and renewable resources of specific regions, and an optimization‐based methodology has been used to quantify the amount of renewable generation capacity needed. Different renewable energy technologies have been considered, in order to meet specific requirements of grid integration of renewables at different horizons of time, up to 100% in the most futuristic case.

The optimization algorithm was exampled in three scenarios, considering and not considering storage. It has been shown that some technologies can provide the renewable backbone (solar PV and wind), but they lack the flexibility needed to achieve a very high share in the energy mix. Other technologies become important to cover the last range of integration (for instance, solar thermal and pumped hydro), as they provide high flexibility, which is crucial for high share, but they are expensive for low share. Otherwise, if these technologies are excluded, the required installed capacity might rise substantially for high constraints of a renewable share if only wind and PV are considered. This reveals that extreme cases in terms of shares of RES might be challenging or even prohibitive if storage is not considered. Therefore, the scenarios discussed in this paper are useful to highlight these future challenges related to the system adequacy and network investment, required to run such RES‐dominated systems.

A number of simplifications were performed in this study, such as the lack of grid constraints, battery storage, and demand‐side management. However, the challenges showed, even with the limited generation mix analyzed, are still revealing the future system needs.

The proposed scenarios can be considered realistic in the sense that real data and real locations inspire them, but they are not detailed in the sense of power system operation, rather serving as a starting point for future studies. As the current power system still contains a large amount of conventional thermal power plants, the current network configuration and the presence of renewable power plants might be subject to important changes over the next years and decades. Further work is being developed by the authors in order to include the network topology and its limitations in the optimization, which will provide more accurate results about specific power systems.

Based on the optimization results applied to the analyzed scenarios, the future European targets that consider a generation mix mainly composed of renewable generation will invariably require the participation of storage technologies in the grid to reduce the ratio between installed RES capacity and maximum demand and increase the system flexibility.

## Conflict of Interest

The authors declare no conflict of interest.

## Data Availability

The data used in this study was derived from the following resources available in the public domain: [https://www.esrl.noaa.gov/gmd/grad/solcalc/calcdetails.html]; [https://www.esios.ree.es/en];[https://www.renewables.ninja/].
